# The adverse health effects of waterpipe smoking in adolescents and young adults: A narrative review

**DOI:** 10.18332/tid/142521

**Published:** 2021-10-22

**Authors:** Olorunfemi Adetona, Sarah Mok, Jenna Rajczyk, Marielle C. Brinkman, Amy K. Ferketich

**Affiliations:** 1 Division of Environmental Health Sciences, College of Public Health, The Ohio State University, Columbus, United States; 2 Division of Epidemiology, College of Public Health, The Ohio State University, Columbus, United States

**Keywords:** waterpipe, hookah, tobacco, young adults, adolescents

## Abstract

Waterpipe (WP) smoking has rapidly grown in popularity in the United States and other Western countries with the fastest uptake among younger individuals. This growth has been encouraged by the misperception that WP smoke is harmless or less harmful than cigarette smoke. To better understand how WP affects the health of young people, we conducted a narrative review of the literature focusing on the adverse health effects of WP smoking in adolescents and younger adults. We searched scientific literature databases including PubMed, MEDLINE, EMBASE, and ISI Web and selected papers that met the inclusion criteria. Sixty-three papers met the inclusion criteria and were selected for review. Data were abstracted from the selected papers into a standardized table. The evidence demonstrates that WP smoking can cause acute lung infection and injury, and carbon monoxide (CO) poisoning, in adolescents and young adults. It is also associated with adverse subclinical effects in this sub-population, including oral and systemic genotoxicity, lung function decline, and the alteration of vascular and hemodynamic functions. Limited evidence that is available indicates associations with psychological and neurological effects and asthma. No identified publications examined the association between WP use and type 2 diabetes, a condition that is associated with cigarette smoking among young people. WP smoking by younger individuals can result in their hospitalization due to systemic CO poisoning and acute lung disease, and induce subclinical adverse effects in the oral cavity, pulmonary system, and in circulation, that are involved in the pathogenesis of local and systemic chronic diseases.

## INTRODUCTION

Also referred to as hookah, shisha, narghile, and hubble-bubble^1^, waterpipe (WP) smoking was traditionally associated with men in the Middle East^2^. However, its use has rapidly expanded to become a global phenomenon over the last three decades^2^. Much of the global growth in WP use is attributable to its relative popularity among youths and young adults^2-4^. For example, 47.5% and 9.2% of young adults (aged 18–24 years), versus 15.5% and 1.2% of adults (aged ≥25 years), in a 2015–2016 national US survey reported ever or current (use within the past 30 days) WP use, respectively^5^. Additionally, prevalence of ever or current use among middle and high school students in the National Youth Tobacco Survey (NYTS) in the US was 7.6–14.6% and 2.5–6.4%, respectively, between 2011 and 2017^6^.

**Figure 1:**
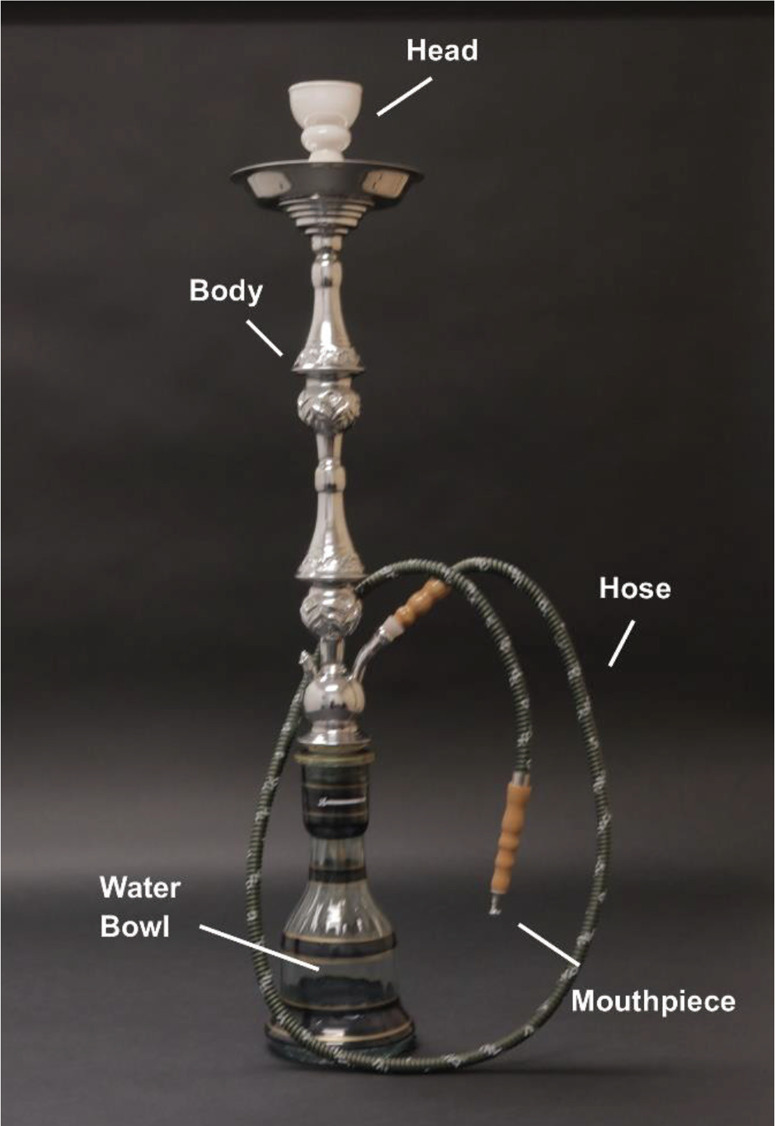
A waterpipe (the charcoal, for heating, and foil are placed on top of the tobacco in the head, but are not pictured)

The global expansion of WP use has been aided by the social context surrounding WP smoking, the availability of flavored WP tobacco, Internet and social media messaging, and the lack of WP-specific tobacco regulations^2^. The growth in its acceptance among younger individuals has also been encouraged by misconceptions about its harmfulness^3,4,6^. Many WP tobacco smokers believe that it is harmless or at least less harmful and less addictive than cigarette smoking^1,4^. As can be seen in Figure 1, WPs involve smoking tobacco that is heated by charcoal in the WP head. When a user sucks on the mouthpiece of the hose that is attached to the WP, the resulting smoke bubbles through water before inhalation. Many WP tobacco smokers believe that the water in the bowl filters out the toxic agents in tobacco smoke^1,4^. The combination of tobacco flavoring and water in the WP bowl results in a humid, milder smoke that is less irritating compared to cigarette smoke^1,4^.

The lower irritation potential of WP tobacco smoke and the significantly lower draw resistance of the WP, compared to other combustible tobacco products, encourage deeper inhalation and longer smoking sessions^1^. The volume of smoke inhaled during a 1-hour session of WP smoking is about 200 times the volume that is drawn from smoking one cigarette^7,8^. Also, one WP smoking session can yield two, five and ten times the amount of nicotine, tar, and carbon monoxide (CO), respectively^7-9^, and produces larger amounts of heavy metals, polycyclic aromatic hydrocarbons, and semi-volatile furans compared to yields from smoking one cigarette^9-11^. Therefore, the associations between WP smoking and chronic respiratory diseases, cardiometabolic diseases, lung cancer, oral and gastrointestinal cancers, and other cancers are not unexpected and are similar to the adverse health effects of cigarette smoking^12,13^.

Although, most of the global growth in the popularity of WP smoking has been among youth and young adults, few studies of its adverse health effects have been conducted among these age groups. We reviewed the available evidence of the health impacts of WP use among youth/young adults (aged 14–34 years, following the US Census categorization of young adults)^14^. While related reviews including recent publications have been conducted^12,13,15-17^, none has focused on adverse effects observed among younger individuals.

### Literature search

Since there are a limited number of studies of the adverse health effects of WP smoking among adolescents and young adults, all epidemiological studies, including case studies, were included in this narrative review. Reviewed studies met the following criteria: 1) must include a WP smoking group and some quantification (at least binary yes/no) of WP smoking; 2) must have collected information about adverse physiological, subclinical or clinical outcomes from study participants; 3) must have determined the association between WP smoking and the adverse health outcome; 4) must have reported the association between WP smoking and the health outcome(s) specific to a study group that is all or mostly adolescents/youths/young adults; and 5) must have been published in English.

Four scientific research databases including PubMed, MEDLINE, EMBASE, and ISI Web Science were electronically searched between April and June 2020 for potentially eligible peer-reviewed articles. The keywords used consisted of synonyms of WP, smoking-related health effects, and terms identifying the age group of interest as outlined below:

- Keyword Group 1 (WP synonyms): waterpipe, hookah, narghile, shisha;
- Keyword Group 2 (Smoking-related health effects): health effects, oral, infection, pulmonary, respiratory, lung function, cardiovascular, cognitive, neurological, psychological, developmental, oxidative stress, inflammation, DNA damage, genotoxicity, cancer, leukemia, sarcoma; and
- Keyword Group 3 (Age group terms): adolescent, youth, young adult.

Searches were made using all combinations of the keywords from the three groups with one keyword from each group. For example, [‘waterpipe’, ‘cardiovascular’, ‘adolescent’] was one of the search strings. The search strings did not include Boolean operators. A total of 555 unique results were identified after duplicates were removed.

One reviewer screened the identified paper for selection, according to the outlined criteria. The selections were then reviewed by another reviewer. Data were abstracted from each selected paper by three of the reviewers into a standardized form (Tables 1 to 5). The abstracted data were thereafter reviewed by one of the reviewers for correctness.

**Table 1:** Studies of oral effects of WP smoking in adolescents and young adults

*Authors Year*	*Study design*	*Study participant characteristics*	*Exposure measures*	*Outcomes of interest*	*Key results*	*Methodological information*
Arazi et al.^25^ 2019	Cross-sectional	N=23 (11 WPS, 12 C); 23.63±2.90 years for WPS, 22.66±2.90 years for C; 100% female - all sedentary	WP smoking	Saliva biomarkers pre-post aerobic exercise (AE)	C > WPS for saliva flow rate (SFR) at all times (SFR only declined immediately after AE in WPS); C > WPS for increase in peroxidase and decrease in DPPH activities immediately after AE; no differences for uric acid.	Self-reported WPS; convenience sample; small sample size and no control for confounding –participants in both groups were similar in age, height, weight, and BMI.
Dehghan Nezhad et al.^21^ 2020	Cross-sectional	N=60 (30 WPS and 30 C); 20–50 years (WPS 26.83±3.74 years with 25 being 20–30 years and 5 being 30–40 years and C 28±7.88 years with 23 being 20–30 years and 5 being 30–40 years); 100% males; Tehran, Iran	WP smoking; WP smoking duration and frequency	Genotoxicity in buccal cells	Micronucleus frequency – WPS > C; WP smoking frequency associated with micronucleus frequency.	Self-reported exposure; controlled for possible confounding by excluding persons that were farmers, worked in arsenic industries, ever smoked > 100 cigarettes, had history of drug use, had dental radiation, had beam exposure, had systemic disease, or had oral legions); persons in WPS and C groups not from the same population.
Eker et al.^22^ 2016	Cross-sectional	N=60 (30 WPS and 30 C); 18–25 years; Sex distribution of both groups were similar; Turkey	WP smoking	Genotoxicity	WPS > C for micronuclei and binucleus cell frequencies.	Self-reported exposure; participant selection approach not stated; no stated control of confounding.
Jafari and Bigdoli^24^ 2017	Cross-sectional	N=20; 25.2±3.67 years; 60% male; Tehran, Iran	WP frequency	Genotoxicity in buccal cells	Micronucleus frequency associated with frequency of WP use (p=0.021).	Self-reported WP smoking; convenience sample; no control for confounding and no true control participant.
Khemiss et al.^30^ 2016	Cross-sectional	N=120 (60 WPS, 60 CS); 28±4 years for WPS, 27±3 years for CS; 100% male; Tunisia	WP smoking	Periodontal status (plaque index, periodontal bone height [PBH])	WPS > CS for plaque index (1.84±0.73 vs 1.54±0.70); PBH similar for both groups	Self-reported WPS; convenience sample; no totally non-exposed control; exclusion based on less tobacco use, morbidity, teeth <20, radiation treatment and medication use; matched based on age, tobacco use, tooth brushing frequency.
Khemiss et al.^26^ 2017	Cross-sectional	N=72 (36 WPS, 36 C); 23±4 years, 22±3 years; 100% males; Tunisia	WP smoking	Saliva physiological and biochemical parameters	Baseline pH and saliva flow rate similar for both groups; WPS<C for buffering capacity.	Self-reported WPS; convenience sampling; exclusion based on less tobacco use, morbidity, radiation treatment and medication use, intra-oral appliances.
Khemiss et al.^29^ 2019	Cross-sectional	N=144 (74 WPS, 74 CS); 100% male; Tunisia	WP smoking	Periodontal status (gingival index, plaque index, decayed/missing/filled teeth [DMFT], probing pocket depth, tooth mobility, periodontal disease, dentist visits)	WPS<CS for gingival index, probing pocket depth and periodontal disease; both groups similar for DMFT, plaque index, tooth mobility and number of missing teeth.	Self-reported WPS; convenience sample; no totally non-exposed control; exclusion based on less tobacco use, morbidity, teeth <20, radiation treatment and medication use; matched based on age, tobacco use, tooth brushing frequency.
Rajabi-Moghaddam et al.^23^ 2020	Cross-sectional	N=90 (30 WPS, 30 CS, 30 C); 24.7±6.3 years for WPS, 23.8±6.9 years for CS, 22.6±5.3 years for C; 100% male; location unspecified	WP smoking	Genotoxicity (buccal cells)	WPS >CS >C for number of micronuclei; WPS >C for number of cells with micronuclei (MNF: micronucleus frequency)	Self-reported exposure; groups matched by age and sex, exclusion of persons with visible lesions or history of malignancy, chronic systemic disease, use of alcohol and other substances.
Seifi et al.^18^ 2014	Cross-sectional	N=120 (40 40 WPS, 40 CS and 40 C); 20–40 years (WPS 30.15±6.02 years, CS 30.32±5.69 years, C 30.3±5.83 years) ; 100% males; Babol, Iran	WPS smoking	Cytological examination of oral mucosa Oral candida Oral cavity inflammation	Nuclear size – CS >WPS >C in buccal, tongue and mouth floor mucosa; cytoplasm size – CS <WPS <C in buccal, tongue and mouth floor mucosa; nuclear/cytoplasm size ratio CS > WPS > C in buccal, tongue and mouth floor mucosa; ferret ratio – CS > WPS > C in buccal, tongue and mouth floor mucosa; no differences in karyorrhexis, vacuolization or cells with multilobular nuclei. Higher percentage of WPS + CS compared to C in buccal, tongue and mouth floor mucosa. Higher percentage of WPS + CS compared to C in buccal, tongue and mouth floor mucosa.	Self-reported exposure status; convenience sampling, groups from different populations; control of potential confounders including age and sex via matching and restriction via eligibility criteria; WPS and CS groups from populations that are different from C group.
Shakhatreh et al.^27^ 2018	Cross-sectional	N=100 (59 WPS, 41 C); 23.98±2.77 years for WPS, 24.14±4.37 years for C; 74.5% and 65.9% male for WPS and C resp.; Irbid City, Jordan	WP smoking	Sub-gingiva and oral cavity microbial flora	Sub-gingiva – WPS > C for frequency of Candida Albicans; Acetinobacter and Moraxella spps. only present in WPS; WPS < C for frequency of Fusobacterium Nucleatum. Oral cavity – WPS > C for CFU of black pigmented bacteria. No differences with Campylobacter, Viridians group Streptococci, Enterobacteriaceae, Staphylococcus Aureus WPS > C for self-reported oral infection.	Self-reported WPS; convenience sample; exclusion based on bleeding gum, oral disease, medication.
Silveira et al.^19^ 2018	Cross-sectional	N=80 (40 WPS, 40 C); 22.55±3.02 years for WPS, 20.00±3.15; 50% male for both groups	WP smoking	Genotoxicity as micronucleus cytome assay (in buccal cells)	Karyolitic cells, karyorrhetic cells, micronuclei, pyknotic cells, binucleated cells, cells with nuclear buds – WPS > C 1.29x, 1.58x, 5.54x, 2.00x, 2.17x, 3.87x respectively; differentiated cells – WPS < C 0.94x.	Self-reported exposure; participant selection unspecified; participants matched on age, gender and alcohol use with control for age and gender; CS and ex-smokers excluded.
Taghibakhsh et al.^20^ 2019	Cross-sectional	N=72 participants (36 WPS and 36 C); mean age 27.3±5.9 years in WPS group and 29.9±6.1 in C; 100% male; Tehran, Iran	WP smoking	Genotoxicity in buccal cells	WPS > C for micronuclues, karyorrhexis, karyolysis and broken eggs with mean frequencies 1.8–2.5 folds of C group; WPS < C for repair index at 0.6-fold of C.	Self-reported exposure; sampling technique not described except stated as ‘objective-based sampling’; excluded persons who smoked cigarettes, consumed alcohol and/or drugs, had systemic disease, exposed to chemical agents, had radiotherapy for head and/or neck; did not control for confounders otherwise.
Al-Belasy^31^ 2004	Prospective cohort	N=300 (100 each of WPS, CS, C); 22–39 years for WPS, 20–38 years for CS, 20–37 years for C; 100% male; Egypt	WP smoking and WP frequency	Dry socket on third molar tooth removal	WPS, CS > C for incidence of dry socket; dose response relationship observed with regards to number of WP sessions per day.	Self-reported WPS, convenience sample but from same dentist practice; excluded patients taking/needing antibiotic prior to procedure; did not control for other potential risk factors for dry socket.

*WPS: waterpipe smokers. CS: cigarette smokers. C: control.

We identified a total of 65 papers that satisfied the selection criteria. Twelve of the studies reported on oral health effects, 16 on pulmonary health effects, 18 on cardiovascular health effects, 10 on psychological/neurological health effects, and 16 on general physiological responses. Of the 65 papers, 52 were either cross-sectional or pre-post studies, 10 were case studies, and 3 were cohort studies. Forty-one of the studies were conducted in the Middle East, 4 in Europe, 14 in North America, 1 in South America, 2 in Asia, and 1 in the Pacific.

## DEVELOPMENTS

### Effects in the oral cavity

The following effects of WP smoking have been studied in the oral cavity in young adults: physiological and biochemical changes in saliva^18,19^; inflammation and cytological effects including chromosomal aberrations in the oral mucosa^20-26^; oral infection and alteration of the oral microbiome^24,27^; and impairment of periodontal health^28-30^ (Table 1). The evidence indicates that WP smoking impairs oral health among young adults, and the demonstration of its genotoxicity in oral mucosal cells is consistent across studies. WP smoking may also alter saliva biochemistry and the oral microbiome, and results suggest that it is associated with tooth and periodontal diseases.

#### Genotoxicity

Indicators of cell death (apoptosis and necrosis) including pyknosis, karyorrhexis, and karyolysis are consistently higher in buccal cells of WP smoking young adults compared to non-smoking controls^24-26^. Similar results have been observed for markers of DNA damage including binucleation, micronuclei, broken eggs, nuclear buds, and chromosome breakage, and chromosome/chromatid fragments and gaps^20,21,23,25,26^. In one study, a greater degree of DNA damage as indicated by micronucleus frequency in exfoliated buccal epithelial cells was observed among persons who exclusively smoked WP compared to those who exclusively smoked cigarettes^23^. Also, micronucleus frequency was associated with the frequency of WP smoking among young adults in a study that did control for cigarette smoking^22^. However, repair index, which was assessed as the ratio of nuclear changes that are evident of both apoptosis and necrosis (karyorrhexis and karyolysis) to nuclear changes that are evident of damage (broken eggs and micronuclei), was 0.6-fold lower in the buccal cells of WP smokers compared to non-smokers^26^. Furthermore, cytometric alterations indicating premalignant and malignant lesions including changes in nuclear size and shape, and increase in the nuclear to cytoplasm ratio were observed in buccal, tongue and mouth floor mucosa cells of WP smokers compared to non-smoking controls. These observations in WP smokers were accompanied by increased inflammation in cytological samples of the mucosa cells of the three oral areas^24^.

#### Effects on saliva biochemistry and oral microbiome

Changes in saliva that can impair oral health have been observed in WP smokers^18,19^. Although, stimulated saliva pH was similar, its buffering capacity was lower among young adult WP smokers compared to age-matched non-smoking controls^19^. This suggests a WP smoke-mediated impairment in the capacity of saliva to protect against enamel demineralization and dental caries^19^. Also, results indicate reduced saliva antioxidant capacity in WP smokers^18^. Peroxidase and 2,2-diphenyl-1-picryl-hydrazyl-hydrate (DPPH) activities were less increased and more reduced, respectively, in WP smoking young adults following exhaustive acute exercise, which is an oxidative stress inducing activity^18^.

WP smoking may also alter the oral microbiome^27^. The frequency of detection of *Candida albicans*, the fungus that causes oral thrush, was higher in the subgingival plaque of young adult WP smokers compared to age-matched non-smoking controls. *Acinetobacter* and *Moraxella spp*. in the subgingival plaque were only detected in the WP smokers, while the colony forming unit (CFU) of black-pigmented bacteria *Porphyromonas gingivalis* and *Prevotella intermedia* was higher in their oral cavity samples (cheek, teeth, and tongue). Some *Acinetobacter* and *Moraxella* species cause respiratory airway infections, while both black-pigmented bacteria species are periodontal pathogens^27^. Meanwhile, the detection frequency of *Fusobacterium nucleatum*, a ubiquitous and mostly commensal microbe in the oral cavity, was lower in the subgingival plaque of the WP smokers^27,31^. The frequency of candida detection in cytological smears of the cheeks, tongue and floor of the mouth of WP smokers was similar to the frequency among cigarette smokers, but lower than the observations made among age- and sex-matched non-smoking young adult controls in another study^24^.

#### Tooth and periodontal effects

Khemiss et al.^29^ reported similar (decayed/missing/filled teeth, plaque index, and tooth mobility) or better (gingival index, probing pocket depth, and periodontal disease) degrees for indicators of teeth and gum health in young adult WP smokers compared to cigarette smokers. However, the same authors had observed higher plaque index in WP smokers in a prior study^30^. Neither study included non-smoking controls for comparison. In another study, young adult WP smokers had a similar likelihood of developing an occurrence of oral dry socket (a very painful condition caused by the dislodgement of the clot and the disruption of the healing at a tooth extraction site) after the extraction of the third mandibular molar tooth compared to cigarette smokers, and a three times higher likelihood compared to non-smokers^28^. The incidence of oral dry socket also increased with smoking frequency, and the outcome was five times more likely to be observed in young adults who smoked WP twelve times per day compared to those who smoked three times per day^28^.

#### Comments about studies of effects in the oral cavity

All the studies of the adverse oral effects of WP smoking in young adults included age- and sex-matched positive and/or negative controls, i.e. cigarette smoking and/or non-smoking groups, which sometimes excluded males or females. However, differences in outcomes based on sex were not investigated in any of the studies. Most of the studies excluded persons with chronic systemic disease or previous oral infection or disease, use of medication and/or radiation exposure. However, there was potential for residual confounding of the results by factors such as consumption of alcohol and confections, environmental chemical exposures, and oral hygiene. All but one of the studies were conducted in Middle Eastern countries^25^. Smoking was self-reported and not objectively measured in all the studies. Lastly, dose-response relationship was assessed in only two studies with an observation that micronucleus frequency in the oral cavity increased in association with a higher frequency of WP usage^20,22^.

### Pulmonary effects

Study results (Table 2) indicate that WP smoking is associated with changes in the molecular milieu (genetic material) and cellular composition in the lungs, decrements in lung function, and respiratory symptoms among adolescents and/or young adults.

**Table 2:** Studies of respiratory effects of WP smoking in adolescents and young adults

*Authors Year*	*Study design*	*Study participant characteristics*	*Exposure measures*	*Outcomes of interest*	*Key results*	*Methodological information*
Alaska^44^ 2019	Case study	22-year-old male; Saudi Arabia	WP smoking	Spontaneous pneumomediastinum	Pneumomediastinum after smoking WP for the first time.	Case study
Annakkaya et al.^46^ 2018	Case study	22-year-old male; Turkey	WP smoking	Acute eosinophilic pneumonia	Acute eosinophilic pneumonia consequent upon WP smoking for the prior 2 months.	Case study
Brosh-Nissimov et al.^48^ 2019	Case study	29-year-old male; Israel - subject had travelled to Goa, India	WP smoking	Respiratory infection	Meliodiosis – chronic cavitary pulmonary infection due to B. pseudomallei suspected from using well water for WP smoking.	Case study
Choe et al.^45^ 2018	Case study	19-year-old male; New Zealand – subject moved from Fiji	WP smoking	Granulomatous lesions in the lungs	Lesions observed consequent upon following regular WP smoking during prior 3 months; resolution of lesions after smoking cessation.	Case study
Kang et al.^47^ 2016	Case study	male in 20s; United States - subject moved from Saudi Arabia	WP smoking	Diffuse lung opacity	Acute eosinophilic pneumonia characterized by progressive lung opacity, inflamed mucosa with accumulation of eosinophils; tachycardia, tachypnea, high WBC count.	Case study
Marchetti et al.^49^ 2020	Case study	20-year-old male; Switzerland	WP smoking	Respiratory infection	Infection with M. tuberculosis after regular WP smoking with no other risk factor present.	Case study
Hawari et al.^38^ 2013	Cross-over	N=24 (24 WPS); all participants 18–26 years (mean 20.4 years); 100% male; Jordan	WP smoking session	Spirometry, cardiopulmonary exercise test	FEF25-75%, baseline respiratory rate, Borg scale at mid and peak exercise increased post WPS session.	WP smoking session; convenience sample; excluded participants with abnormal cardiovascular health measures, fever, acute upper or lower respiratory tract infections.
Fedele et al.^50^ 2016	Cross-sectional	N=32921; all participants 9th–12th grade; 51.3% male; 39.1% White, 22.3% Black, 30.6% Hispanic, 8.0% Others; Florida	WP Smoking	Self-reported asthma	OR=1.32 of current use of WP among asthmatics.	No control for confounders, self-reported WP smoking and outcome.
Hawari et al.^39^ 2017	Cross-sectional	N=138 (69 WPS and 69 C); all participants 18–26 years (mean 22.1 years in WPS group and 21.4 years in C group); 100% males; Jordan	WPS smoking	Respiratory – symptoms, lung function	WPS > C for reported respiratory symptoms; WPS < C for FEV1, FVC, PEF and TLC.	Self-reported exposure; convenience sample used; relatively small sample size; excluded participants based on BMI, active chronic medical conditions, chronic prescription medications use, illicit drug use, cardiovascular health measures, oxygen saturation; controlled for factors influencing exercise duration in analysis.
Hawari et al.^42^ 2019	Cross-sectional	N=738 (135 WPS, 303 CS, 300 C); 18–27 years (21.5±1.8 for WPS, 21.7±1.9 for CS, 21.6±1.9 for C); 100% male; college campuses in Egypt, Jordan, Morocco, Oman	WP smoking, WP duration, WP frequency	Respiratory symptoms	Prevalence rate of respiratory symptoms – WPS = 1.6x of C and C = 1.9x of C; WPS or CS > C for prevalence rate of cough or phlegm.	Self-reported exposure; self-reported outcome; convenience sample; controlled for environmental exposures, BMI, physical activities, excluded persons with chronic diseases.
Husain et al.^37^ 2016	Cross-sectional	N=525 (52 WPS, 69 CS, 122 WPS + CS, 282 C); 16–32 years (20.7±2.1); 100% male; multiple universities in Kuwait	WP smoking; WP frequency	Cardiorespiratory health Lung function (peak expiratory flow rate – PEFR)	Persistent cough, chest pain, rapid heart rate –WPS < CS but not different from C. No significant difference for respiratory infections, shortness of breath, high BP, increase blood sugar levels, sleep disturbances. PEFR - WPS or CS > C, WP > CS but not statistically significant.	Self-reported exposure only; convenience sample used, no control for confounding.
Martinasek et al.^51^ 2013	Cross-sectional	N=36578 (4905 WPS – 57.1% White, 5.3% Black, 27.2% Hispanic, 10.4% Others); all participants 9th–12th grade; 51.6% male; Florida	WP Smoking	Self-reported asthma	Higher prevalence of ever and current WP users among lifetime and current asthmatics (p<0.05).	Descriptive statistics, no control for confounders, self-reported WP smoking and outcome.
Meo et al.^34^ 2014	Cross-sectional	N=146 (73 WPS and 73 C); mean age – 21.54±0.41 years in WPS group and 21.36±0.19 in C; 100% male; Riyadh, Saudi Arabia	WP smoking	Lung function (via spirometry) Respiratory inflammation (Fractional Exhaled Nitric Oxide [FENO] concentration)	WPS < C for lung function parameters (FEV1, FEV1/FVC ratio, FEF-25%, FEF-50%, FEF-75% and FEF-75-85%. WPS < C for FENO.	Self-reported exposure; convenience sample; excluded persons with chronic systemic diseases, substance users, tobacco smokers (other than WP), regular vigorous exercise, potential confounding occupational exposure; age, height, and weight were similar between groups; did not control for confounding otherwise.
Strulovici-Barel et al.^33^ 2016	Cross-sectional	N=40 participants (19 non-smoker C and 21 WPS); all participants ≥18 years (mean 33±9 years in C group and 25±4 years in WPS group); 90% male in C, 65% male in WPS group; 6/5/8 black/white/other in C group and 8/2/11 black/white/other in WPS group; New York City, USA	WPS smoking	Respiratory symptoms Metabolomic profile of respiratory tract epithelial lining fluid Cellular composition in small airway epithelium (SAE) Transcriptome of SAE and alveolar macrophages (AM)	WPS > C for cough and sputum scores. 31 (out of 1675) features with significantly different abundance in WPS vs C. WPS > C for secretory cells; WPS < C for basal and ciliated cells. 159 differentially expressed genes in SAE, 181 differentially expressed in AM in WPS vs C; WPS > C for transcriptome response score for both SAE and AM.	Self-reported exposure verified with cotinine and nicotine measurements; convenience sample used; no confounder control but study participants in C group and WPS group were comparable in terms of sex, ethnicity, body mass index, and alpha-1 antitrypsin levels.
Tamim et al.^43^ 2003	Cross-sectional	N=625 (53 WPS, 270 CS; 115 WPS + CS, 187 C); 10–15 years; 51.6% male; Beirut, Lebanon	WP smoking by adult household member	Respiratory symptoms	OR (CI): wheezing or nasal congestion for WPS – 2.3 (1.1–5.1), wheezing or nasal congestion for WPS + CS – 3.0 (1.7–5.6), wheezing for WPS + CS – 4.9 (2.3–10.7), nasal congestion for WPS + CS – 2.3 (1.1–4.8) compared to C.	Self-reported (presence/absence) exposure only; participant selection process unspecified; no control for confounding.
Walters et al.^32^ 2017	Cross-sectional	N=14 (7 WPS, 7 C); 27±9 years for WPS, 30±4 years for C; 3 males in each group; black/white/other distribution – 3/0/4 for WPS, 3/1/3 for C; New York, USA	‘Light’ WP smoking (<5 sessions/week)	Epigenetic and transcriptomic changes in small airway epithelial cells	PCA of all DNA methylation probe sets showed separation of samples by WP smoking status; 727 differentially methylated probe sets (>1.5-fold change) representing 673 genes; 11.3% of these had significant change in gene expression.	Details about sample selection limited and small sample size; included healthy subjects (defined on basis of physical exams, physiological and biochemical parameters, and medical history); age, gender, ethnicity and region of SAE controlled for in PCA.
Yalcin et al.^36^ 2017	Cross-sectional, pre-post	N=100 (50 WPS/WPS + CS and 50 C) 18–38 years (26.72±5.2 years for WPS, 27.46±5.3 years for C); 66% male for WPS, 64% male for C; Ankara, Turkey	WP smoking; WP duration; exhaled CO	Lung function (spirometry)	FVC% and FEV1% (WPS + CS < WPS, C); FEV1/FVC (WPS + CS < C); FVC%, FEV1%, FEV1/FVC, FEF25-75 reduced after WP smoking.	Controlled WP smoking session; objective exhaled CO used to quantify exposure but association with outcome not analyzed; convenience sample used; no control for confounding.

*WPS: waterpipe smokers. CS: cigarette smokers. C: control.

#### Molecular and cellular effects

Associations between WP smoking and epigenetic modifications, which in some cases overlapped with differential gene expression (mRNA transcripts), have been observed^32^. Walters et al.^32^ reported that there was at least a 1.5-fold difference in the degree of methylation of the DNA in 623 unique genes in the cells of the small airway epithelium (SAE) of ‘light’ (≤5 sessions per week) young adult users of WP, relative to non-smoking controls. Xenobiotic metabolism and cellular signaling (e.g. aryl hydrocarbon and G-coupled receptors), which are associated with cigarette smoking and smoking-associated pulmonary disease, were among the biological pathways that were mostly impacted by the observed differential methylation. Furthermore, there were differences in the expression of 11.3% of the differentially methylated genes between the WP users compared to the non-smokers. Differential expressions in 159 and 181 genes in SAE and alveolar macrophage (AM), respectively, were associated with WP smoking (light users) in another study that was conducted by the same research group.

While there was some overlap between WP and cigarette smokers in the cellular pathways that were affected by differential methylation (xenobiotic metabolism, and aryl hydrocarbon and G-coupled receptor signaling), other impacted pathways were unique to WP smokers^32^. Additionally, there was a predominance of hypermethylation in affected genes in SAE among WP smokers compared to non-smokers, whereas hypomethylation was predominant in cigarette smokers^32,33^. There was also little overlap in the differentially expressed genes in SAE between WP and cigarette smokers relative to non-smokers. These observations suggest differences in pulmonary pathology that is potentially driven by the differences in the emission contents of tobacco combustion between WP and cigarette smoking.

Similar to cigarette smokers, the composition of recovered SAE cells was altered in young adult WP smokers when compared with non-smokers^33^. However, the pattern of the alteration was different between the two smoking groups. While there was an increased proportion of secretory cells and a decreased proportion of ciliated cells in recovered SAE cells in both groups compared to non-smokers, WP smokers, unlike cigarette smokers, had an increased proportion of basal cells that are the progenitor cells in the airway epithelium^33^.

#### Effects on pulmonary physiology

Meo et al.^34^ observed a significant decrease in fractional exhaled nitric oxide (FENO) among young adult WP smokers compared to non-smokers. This result indicates the potential oxidative effect of WP smoking in the respiratory airways, possibly partly due to the conversion of nitric oxide to peroxynitrite by reactive oxygen and nitrogen species released in WP smoke^34^. It also suggests that WP smoking can impair nitric oxide physiological function in regulating both pulmonary function and its bronchodilatory effect which plays an important role in homeostasis and disease^35^. A few studies have reported an effect of WP smoking on pulmonary or lung function among adolescents and young adults^33,34,36-38^. Meo et al.^34^ reported lower values for various spirometry measures including forced expiratory volume in one second (FEV_1_), its ratio (FEV_1_/FVC) to forced vital capacity (FVC), and forced expiratory flows (FEFs) at different percentages (25, 50, 75, and 75–85%) of FVC among WP smokers compared to non-smoking young adults. Additionally, peak expiratory flow (PEF) was lower among young tobacco smoking adults^37^. Although, the types of tobacco smoking were not differentiated in the comparison with non-smokers in the study, PEF was lower (insignificantly) among persons who exclusively smoked WP compared to those who exclusively smoked cigarettes^37^. Lower percentages of predicted spirometry values, especially for FVC, FEV_1_, and PEF, were similarly observed among young adult WP smokers vs non-smokers^33,36,39^. These findings suggest that WP smoking could contribute to obstructive and restrictive lung pathologies and the development of chronic pulmonary disease^40,41^. Furthermore, acute decline in lung function following WP smoking has been demonstrated^36,38^, and this was accompanied by increased breathing rate in one of the studies^38^.

#### Respiratory symptoms, lung injury, lung infection, and lung disease

WP smoke exposure was consistently associated with increased risk of respiratory symptoms across studies^39,42,43^. In a cross-sectional study, Hawari et al.^39^ reported that a significantly higher proportion of young adult WP smokers, compared with non-smoking controls, self-reported any respiratory symptom (72.5% vs 21.7%), chest illness that prevented them from working within the previous three years (25.0% vs 10.1%), and coughing up phlegm lasting more than three weeks (11.6% vs 0.0%). In another study that was conducted across multiple Middle Eastern countries, the same research group observed that college student WP smokers were 60% more likely to report respiratory symptoms, e.g. cough and phlegm, compared to non-smokers^42^. There was no difference in the risk for these outcomes compared to cigarette smokers in both studies. Relative to secondhand smoke exposure, elementary school children who lived at home with at least one person who smoked WP and/or cigarettes, compared with children who did not, had increased incidence of wheezing and/or nasal congestion within the previous year^43^. Consistent with the evidence indicating increased risk of respiratory symptoms, cases of acute lung injury (granulomatous lesions and pneumomediastinum)^44,45^, pulmonary eosinophilic inflammation^46,47^, and pulmonary infection (*mycobacterium tuberculosis* and *burkholderia pseudomallei*)^48,49^ following WP smoking activities have been reported in young adult individuals. However, there was no apparent increase in the risk of respiratory symptoms among WP smoking young adults compared to non-smokers in another study^37^. Finally, higher prevalence or odds of WP smoking was observed among persons with asthma in cross-sectional surveys of 9th to12th graders in Florida^50,51^.

#### Comments about studies of pulmonary effects

All but one (a cross-over study) of the studies of pulmonary effects of WP smoking in adolescents and/or young adults were case studies, pre-post, or cross-sectional studies. Most (66%) of the 15 studies were conducted in the Middle East. Also, most relied on self-reported smoking information, and did not control for potential confounders. Whereas some of the studies included both male and female participants, none of the studies tested gender effects and none provided dose-response information.

### Cardiovascular effects

WP smoke is associated with adverse cardiovascular outcomes^16^. Several studies have reported that WP smoking induces hemodynamic and vascular responses, and impairs cardiac autonomic control in adolescents and young adults (Table 3).

**Table 3:** Studies of cardiovascular effects of WP smoking in adolescents and young adults

*Authors Year*	*Study design*	*Study participant characteristics*	*Exposure measures*	*Outcomes of interest*	*Key results*	*Methodological information*
Ahmadian et al.^84^ 2017	Cross-sectional	N=20 (10 WPS, 10 C) age >20 years (27.6±3.1 for WPS, 26.1±3.6 for C); 100% sedentary male; Aliabad Katoul, Iran	WP Smoking	Hematological parameters	WPS > C for hematological variables (white blood cells, hematocrit, lymphocytes, neutrophils).	Self-reported exposure to WP; participant selection process unspecified, no control for confounding.
Alomari et al.^62^ 2018	Cross-sectional	N=397 (161 WPS, 236 C); 14.5±1.1 years for both WPS and C; 59.6% male WPS, 54.6% male C. Irbid, Jordan	WP smoking; WP frequency	Hemodynamics	Heart rate, DBP, mean arterial BP, and rate pressure product (WPS < C).	Self-reported exposure to WP; convenience sample used; excluded students with hyper – glycemia, tension, lipidemia, choleterolemia; mood disorders, medication that can alter cardiovascular function.
Alomari et al.^63^ 2020	Cross-sectional	N=771 (161 WPS, 69 CS, 295 WPS+CS, 246 C); WPS 14.6±1.1 years; CS 14.6±0.99 years; WPS+CS 14.8±1.0 years, C 14.5±1.1 years); Irbid, Jordan	WP smoking, WP frequency	Hemodynamics	WPS, CS < C for heart rate, DBP, mean arterial BP, and rate pressure product. Results not different when stratified by gender.	Self-reported exposure; convenience sample used; control for confounding including BMI, height, and gender
Al-Safi et al.^61^ 2009	Cross-sectional	N=9648 (N=14310 total) for relevant age group (1803 WPS, 7845 C); 18–33 years; 50% male; all regions of Jordan	WP smoking	Hemodynamics	Diastolic BP, systolic BP, mean arterial BP – WPS > C	Self-reported exposure only, exclusivity of WP or cigarette smoking; convenience sample used; excluded individuals with previously cardiovascular disease.
Hawari et al.^39^ 2017	Cross-sectional	N=138 (69 WPS and 69 C); all participants 18–26 years (mean 22.1 years in WPS group and 21.4 years in C group); 100% males; Jordan	WPS smoking	Cardiopulmonary exercise test	WPS < C for VO2 mL/kg and HR at peak exercise, change in EELV, HRR, Pet CO2, and VE/VCO2; WPS > C for shortness of breath and leg fatigue at mid exercise; WPS < C for exercise time in both bivariate and multivariate analyses.	Self-reported exposure; convenience sample used; relatively small sample size; excluded participants based on BMI, active chronic medical conditions, chronic prescription medications use, illicit drug use, cardiovascular health measures, oxygen saturation; controlled for factors influencing exercise duration in analysis.
Selim et al.^76^ 2013	Cross-sectional	N=70 (30 WPS, 30 CS, 10 C) 25–35 years (28±3 years for WPS, 29±3 for CS and 30±3.6 for C); 100% male; Cairo, Egypt	WP Smoking, WP frequency and duration	Vascular function	Flow mediated dilation (FMD%) – WPS < CS < C; FMD% reduced with increasing duration (year) and frequency per day of WP and for indoor vs outdoor WP smoking.	Self-reported exposure only; convenience sample used; excluded combined smokers, persons with cardiovascular disease, or taking medication affecting vasomotor function.
Alomari et al.^54^ 2014	Pre-post	N=53 (53 WPS) 18–35 years (mean: 22.7±4.8); gender unspecified; Irbid, Jordan	WP smoking session	Hemodynamics	WPS session increased heart rate, DBP, mean arterial BP, rate pressure product, and post-occlusion vascular resistance. Post-occlusion blood flow and venous outflow decreased afterwards.	Controlled WP smoking session; participant selection process unspecified; excluded persons with acute medical conditions, cardiovascular, kidney, or metabolic disease, or those using medications with cardiovascular effect.
Alomari et al.^55^ 2015	Pre-post	N=53 (53 WPS) 18–36 years (22.7±4.8); 64% male; Irbid, Jordan	WP smoking session	Hemodynamics	WPS session slightly decreased forearm post-occlusion blood flow, increased post occlusion vascular resistance, and decreased post occlusion venous outflow.	Controlled WP smoking session; exclusion based on chronic diseases, regular use of prescription medication, pregnancy or breast feeding, cigarette smoking.
Cobb et al.^60^ 2012	Pre-post	N=32 WPS; 21.6±2.7 years; 50% male; 68.9% Non-White; Richmond, VA, US	WP smoking session	Hemodynamics Autonomic cardiac control (frequency domain)	Heart rate (immediately after), DBP (immediately and 15 mins after), and SBP (15 mins after) increased following smoking WP with tobacco but not tobacco-free WP. Heart rate variability measures – low frequency power and its ratio to high frequency power increased, sample entropy decreased immediately after smoking WP with tobacco but not tobacco-free WP.	Required abstinence from WP smoking, confirmed objectively; convenience sample; inclusion based on number of tobacco products smoked, objective cardiopulmonary measures, chronic diseases, pregnancy status.
Kadhum^57^ 2014	Pre-post	N=61 (61 WPS) 18–25 years; 80% male; London, UK	WP smoking session	Hemodynamics	DBP, SBP, mean arterial BP and heart rate increased post-smoking; change across WP smoking session not associated with CO.	Controlled WP smoking session; convenience sample; excluded CS, non-WP smokers, individuals with cardiopulmonary disease.
Nelson et al.^53^ 2016	Pre-post	N=28 WPS; 27±1 years; 71.4% male; 32.1% Non-Hispanic White, 50% Non-Hispanic Black, 17.9% Other; US	WP smoking session	Hemodynamics Myocardial blood flow	DBP, SBP, and mean arterial blood pressures, and heart rate increased significantly after WP smoking. Myocardial blood flow velocity, blood flow, and conductance (1.1–1.5 au/mmHg) increased after WP smoking.	Required abstinence from WP smoking, confirmed objectively; convenience sample; inclusion criteria based on number of tobacco products smoked, objective cardiopulmonary measures, BMI, pregnancy status.
Rezk-Hanna et al.^58^ 2018	Pre-post	N=48 (48 WPS) 18–34 years (25±4); 65% male; Los Angeles, CA	WP smoking session	Vascular function Hemodynamics	Central arterial stiffness (augmentation index) and carotidfemoral pulse wave velocity increased post WP; no gender differences. After smoking, increase in heart rate, respiratory rate, brachial artery and aortic DBP and SBP; no gender differences.	Controlled WP smoking session with objectively measured exposure exhaled CO and plasma nicotine; convenience sample used; exclusion based on chronic systemic disease, drug use, physical evidence of cardiopulmonary disease, sinus rhythm, pregnancy, prescription medication, antioxidant supplementations, pre-smoking exhaled CO ≥10 ppm, psychiatric illness.
Rezk-Hanna et al.^59^ 2019	Pre-post	N=30 WPS, 15 CS; 26±1 years; 62.2% male; 40% Non-Hispanic White, 22.2% Non-Hispanic Black, 7% Hispanic, 20% Asian, 11% Other; US	WP smoking session	Vascular function	FMD – changed by +43±6%, -27±4%, +138±71%, -36±4% after smoking charcoal heated WP, electrically heated WP, 0.1% carbon monoxide, cigarette.	Required abstinence from WP smoking, confirmed objectively; convenience sample; inclusion criteria based on number of tobacco products smoked, healthy cardiopulmonary measures, BMI, pregnancy status.
Rezk-Hanna et al.^52^ 2020	Pre-post	N=21; 24±1 years; 57% male; 38.1% Non-Hispanic White, 33.3% Non-Hispanic Black, 19% Hispanic, 9.5% Other; US	WP smoking session	Hemodynamics Hemodynamics	DBP, SBP, and mean arterial blood pressures, and heart rate increased significantly 30 minutes after WP smoking Foot skin blood flow reduced and vascular resistance increased respectively, calf muscle blood flow and vascular resistance increased and reduced respectively post-flow; changes sustained until 30 minutes after WP smoking.	WP smoking session; required abstinence from WP smoking, confirmed objectively; convenience sample; inclusion criteria based on objective cardiopulmonary measures, BMI, pregnancy status.
Rezk-Hanna et al.^59^ 2019	Pre-post	N=30 WPS, 15 CS; 26±1 years; 62.2% male; 40% Non-Hispanic White, 22.2% Non-Hispanic Black, 7% Hispanic, 20% Asian, 11% Other; US	WP smoking session	Vascular function	FMD – changed by +43±6%, -27±4%, +138±71%, -36±4% after smoking charcoal heated WP, electrically heated WP, 0.1% carbon monoxide, cigarette.	Required abstinence from WP smoking, confirmed objectively; convenience sample; inclusion criteria based on number of tobacco products smoked, healthy cardiopulmonary measures, BMI, pregnancy status.
Yildirim et al.^77^ 2016	Pre-post	N=33 (33 WPS); 26.8±6.2 years; 84.8% male	WP smoking session	Electrocardiogram, hemodynamics	DBP, SBP increased; oxygen saturation decreased; dispersions of QT, QTc, P-wave and Tp-Te increased after WP smoking.	Self-reported WPS; convenience sample; excluded persons aged <18 years, cardiovascular morbidity/medication.
Zhou et al.^56^ 2017	Pre-post	N=10 (hookah bar workers); ≥20 years (mean: 26.6±2.8); 20% male; NYC (Manhattan), NY	Air quality of hookah bar exhaled CO, saliva cotinine	Hemodynamics	Non-statistically significant increase in heart rate, SBP, and DBP across work shift.	Objective exposure measured by air sampling and biomarkers; convenience sample used; inclusion limited to people aged ≥20 years, working in hookah bar, and exclusion of pregnant women, current CS; small sample size.

*WPS: waterpipe smokers. CS: cigarette smokers. C: control.

#### Hemodynamic effects

The acute vascular response to WP smoking among young adults reflects the differential effects of tobacco smoke components in different tissue beds. In one study, calf muscle blood flow and vascular resistance measured by venous occlusion strain-gauge plethysmography increased and decreased, respectively, in young adults following WP smoking^52^. Similarly, Nelson et al.^53^ observed an increase in myocardial blood flow and conductance in young adults following WP smoking. In contrast, blood flow decreased and vascular resistance increased in the forearm and foot skin following WP smoking^52,54,55^. These differential effects are the consequence of the dilatory effect of nicotine in skeletal muscles and coronary vessels versus its constrictive effect in cutaneous vessels^52,53^. Carbon monoxide, another major component of WP tobacco smoke, also has a dilatory effect in the skeletal muscle beds and coronary vessels, but not in the skin^52,53^.

Also, evidence in the literature suggests opposite directionality for the effects of acute and chronic WP smoking on blood pressure. Although, diastolic blood pressure (DBP), systolic blood pressure (SBP) and heart rate (HR) did not change across work shifts among WP bar workers exposed to secondhand smoke^56^, observations about increases in these measures and mean arterial pressure (MAP) among young adults following WP smoking are consistent across studies^52-54,57-60^. Al-Safi et al.^61^ also reported higher SBP, DBP, MAP and HR in young adult WP smokers compared to non-smokers in a large population-based study (n=7845), while WP smoking was not associated with the prevalence of high BP among young adults in another study^37^. However, results in other studies suggest an opposite chronic effect among adolescents with WP smoking-associated decreases in BP being observed only among boys in one of the studies^62,63^. The apparent opposed directions in the BP effect of acute (increased BP) and chronic (decreased BP) WP smoke exposure is similar to what has been reported for cigarette smoke^64-71^. Alomari et al.^64^ observed reduced BP among WP smoking adolescents compared to non-smoking controls.

Although the basis for the seeming dichotomy in BP effects has not been clarified, the intermittent stimulation of the sympathetic nervous system, that is causal for vasoconstriction and increased hemodynamic activity, by repeated exposure to nicotine from tobacco smoking, and subsequent ‘over-compensation’ of the body and excessive vasodilation in the absence of nicotine during non-smoking periods have been hypothesized as a potential mechanism^62-64^. It is important to note that reduced BP is not necessarily beneficial or harmless as it could be a risk factor for cardiovascular (especially coronary) events^62,72-75^.

#### Impairment of vascular function and other cardiovascular effects

WP smoking impairs vascular function in young adults^58,59,76^. A 30-minute smoking session of a standard charcoal-heated WP increased central arterial stiffness as indicated by increased pulse wave velocity and augmentation index^58^. However, charcoal-heated WP smoking in another study induced a contrary effect on endothelial function with flow-mediated dilation (FMD) increasing after smoking^59^. This was attributed to the high content of vasodilatory CO in smoke from the combustion of the charcoal. In the study, inhalation of a CO gas mixture that achieved a similar CO boost as the smoking of the charcoal-heated WP caused a larger increase in FMD. In contrast, FMD decreased after the smoking of an electronically heated WP. Therefore, it was concluded that CO masks the induction of endothelial dysfunction by other components of WP smoke including nicotine and particulates. The authors of the study hypothesized that this attenuating effect is temporary and that FMD will eventually decrease following CO clearance from circulation^59^. Indeed, Selim et al.^76^ reported that FMD was reduced 0.66x and 0.37x in WP smoking young adults compared to cigarette smoking and non-smoking controls, with an inverse relationships between FMD and the number of WP smoking session per day.

Furthermore, the impairment of cardiac autonomic control, which is associated with adverse cardiovascular events, is observed following WP smoking in young adults^60,77^. Also, Hawari et al.^39^ observed that exercise performance, as indicated by components of the cardiopulmonary exercise test (CPET), was impaired among young adult WP smokers compared to non-smokers. Heart rate, perceived exertion (Borg scale), and self-reported leg fatigue were increased, and peak oxygen consumption and exercise time were reduced among the smokers.

#### Comments about studies of cardiovascular effects

All 19 studies of the cardiovascular effect of WP smoking among adolescents and young adults that were identified were either cross-sectional or pre-post in design. Thirteen studies reported on acute effects; twelve studies were conducted in the Middle East, six in the United States, and one in the United Kingdom. Most of the studies controlled for potential confounders by using them as selection criteria of the study. Although only six of the studies were exclusively composed of male participants, or did not report gender distribution, just one study reported on testing for gender differences. Finally, dose-response association was reported by only one of the studies.

### Neurological and psychological effects

In general, tobacco use is linked with the impairment of mental health. However, the etiology and the directionality of the association is not clear^78-80^. Depression, for example, could predispose a person towards tobacco smoking^78-80^. On the other hand, smoking might be a cause of depression^78-80^. A third alternative is that there is not a causal link between the two, and that they both occur due to common risk factors^79^. Nonetheless, there are few studies (Table 4) about the association between WP smoking and mental health disorders among adolescents and young adults, but the results of the studies are inconsistent.

**Table 4:** Studies of neurological and psychological effects of WP smoking in adolescents and young adults

*Authors Year*	*Study design*	*Study participant characteristics*	*Exposure measures*	*Outcomes of interest*	*Key results*	*Methodological information*
Marsden et al.^79^ 2019	Cohort	N=5236 (out of 5482) smokers (N=885 smoked WP); 18–29 years; 36.7% male; 37.3% non-Hispanic white, 30.9% Hispanic, 16.8% Asian, 7.5% Black, 7.5% others; Texas, US	WP smoking; WP frequency	Psychological – depression (CES-D-10)	RR is 1.03 (1.01–1.05) for a 1-unit rise in scaled unit of frequency of WP smoking; RR is 1.04 (1.01–1.06) and 1.09 (1.04–1.15) for 5-day and 15-day of use within last 30 days, respectively.	Large sample of college students from multiple recruitment waves with repeated measurements; information collected at 6-month intervals; adjustments for age, ethnicity, sex, educational level, father’s education and wave number.
Ahmadian et al.^84^ 2017	Cross-sectional	N=20 (10 WPS, 10 C) age >20 years (27.6±3.1 for WPS, 26.1±3.6 for C); 100% sedentary male; Aliabad Katoul, Iran	WP Smoking	Cognitive function – number recall within 30 s Wingate supramaximal exercise test	No significant difference observed between WPS and C pre- and post-exercise.	Self-reported exposure to WP; participant selection process unspecified, no control for confounding.
Alomari et al.^87^ 2018	Cross-sectional	N=483 (195 WPS, 288 C); 14.4±1.1 years; 45.3% male; Irbid, Jordan	WP smoking	Neurological – biomarker (brain-derived neurotrophic factor – BDNF)	WP smoking associated with reduced circulating BDNF	Self-reported exposure; multi-stage random cluster sampling; controlled for BMI, age, gender and location in analysis; individuals self-reporting hyperlipidemia, hypercholesterolemia, hypertension, cardiac condition, hyperglycemia, psychiatric and stress-related mood disorders were excluded.
Bandiera et al.^78^ 2016	Cross-sectional	N=5438 students from 24 metropolitan colleges; 18–29 years; 63.8% female; 36.3% non-Hispanic white, 31.3% Hispanic, 16.9% Asian, 8.1 non-Hispanic Black, 7.5% Other; Texas	WP smoking	Psychological – depression (CES-D-10)	RR for depressive symptoms is 1.01 (0.85–1.19) for WPS use.	Self-reported exposure to WP; not clear if WP smokers were not multi-product users; large sample of college students from multiple colleges; adjustments for age, ethnicity, gender, college type.
Goodwin et al.^81^ 2014	Cross-sectional	N=1799 college students; 20.1±1.5 years for WPS, 19.8±1.4 for C; 58.8% female; Northeastern US	WP smoking	Psychological – mental health (self-reported diagnosis/treatment by a physician), perceived stress (self-reported level)	No association with mental health problems; no association with perceived stress.	Self-reported exposure; adjustments for age, gender, sorority status.
Heinz et al.^82^ 2013	Cross-sectional	N=143 (48% ever WPS); 19.26±3.42 years; 24% male; 36% Caucasian, 7% African American, 19% Hispanic, 33% Asian, 5% Other; Chicago, US	WP smoking, WP frequency	Psychological (depression) – Inventory to Diagnose Depression	No difference in depression symptomatology by WP use.	Self-reported exposure; convenience sample; lack of control for confounders.
King et al.^83^ 2018	Cross-sectional	N=2370; 21.1±0.4 years; 35.9% male; 83.2% White, 16.8% Non-White; Colleges in VA and NC, US	WP smoking, WP frequency	Psychological – self-reported mental health conditions (depression, anxiety, ADHD), stress (Cohen’s 10-item Perceived Stress Scale), depression (CES-D Short Form)	OR (CI) for WP use – 1.04 (1.01–1.06) per increase on Stress Scale Score, 1.03 (1.00–1.07) per increase on Depression Scale Score, no association with mental health diagnosis.	Self-reported WP smoking and mental health diagnoses; survey of college students, controlled for age, sex, race, ethnicity, and mother's education and cigarette smoking.
Meo et al.^86^ 2017	Cross-sectional	N=65 (33 WPS, 32 C); 24.45±2.93 years for WPS, 23.32±2.68 years for C); 100% male; Riyadh, Saudi Arabia	WP smoking	Neurological – cognitive function (Cambridge Neuropsychological Automated Battery – CANTAB)	WPS < C for attention switching task (AST) latency, AST congruent, AST incongruent, mean choice reaction time (CRT), CRT%.	Self-reported exposure to WP; groups matched based on age, gender, ethnicity, weight, height, SES, education level; excluded based on chronic morbidity, substance use and cigarette smoking; convenience sample with groups apparently from different populations; WPS not exclusively WP smoking.
Primack et al.^80^ 2013	Cross-sectional	N=100891; about 92% 18–26 years; 65.7% male; 70.2% White, 4.8% Black, 6.1% Hispanic, 9.7% Asian, 9.2% Other; Colleges in US	WP smoking, WP frequency	Psychological – self-reported mental health conditions (depression, anxiety, sleep disorder, ADHD, addictive disorder, stress)	OR (CI) for WP use – 1.4 (1.3–1.5) if depressed, 1.1 (1.0–1.2) if stressed.	Self-reported WP smoking and mental health diagnoses; large national survey; controlled for gender, sexual orientation, undergraduate status, race, relationship status, region, population size, and clustering by school.
Saadat et al.^85^ 2018	Pre-post	N=22 (WPS and CS – numbers not noted); 18–22 years (mean: 21.4±0.8); 100% male; Tehran, Iran	WP smoking session	Cognitive – psychomotor driving test	Two-hand coordination total mean duration score, but no other score, improved after WP smoking.	Controlled WP smoking session; convenience sample; controlled for order of test; objectively determined and controlled for nicotine dependence.

*WPS: waterpipe smokers. CS: cigarette smokers. C: control.

**Table 5:** Studies of systemic and general health effects of WP smoking in adolescents and young adults

*Authors Year*	*Study design*	*Study participant characteristics*	*Exposure measures*	*Outcomes of interest*	*Key results*	*Methodological information*
Arziman et al.^89^ 2011	Case study	N=4; 17–27 years, age not stated for one; 25% male; Turkey	WP smoking	Systemic CO poisoning	COHb of 11.4–21.3% recorded and case presented with various symptoms including nausea, syncope, sinus and physical tachycardia and vertigo.	Case study
de Suremain et al.^90^ 2018	Case study	13-year-old male; Paris, France	WP smoking	Systemic CO poisoning	COHb of 23.1% recorded and case presented with CO poisoning after WP smoking.	Case study
Lim et al.^91^ 2019	Case study	19-year-old male; Singapore	WP smoking	Systemic CO poisoning	COHb of 27.8% recorded and case presented with CO poisoning after WP smoking.	Case study
van Rappard et al.^92^ 2014	Case study	N=4; 16–21 years; 50% male; Germany	WP smoking	Systemic CO poisoning	COHb of 16.7–29.6% recorded; 1 asymptomatic, 3 symptomatic presenting with various symptoms including nausea, syncope, headache and paresthesia.	Case study
Alomari et al.^95^ 2018	Cross-sectional	N=475 (WPS 93, CS 44, WP+CS 173, C 165); 12–17 years (mean: 14.6±1); 55% male; Irbid, Jordan	WP smoking	Serum vascular endothelial growth factor (VEGF)	VEGF (WPS + CS < WPS < CS or C) – result due to difference in boys.	Self-reported exposure only; convenience sample used; control for confounding by age, gender, location and BMI in statistical analysis.
Alsaad et al.^93^ 2019	Cross-sectional	N=45 (15 WPS, 15 CS, 15 C); 18–40 years (≥80% of all groups 20–29 years); Riyadh, Saudi Arabia	Frequency of WP smoking	General - oxidative stress, DNA repair gene expression, xenobiotic activating and detoxifying Enzyme gene expression	Blood 8-OHdG (CS > WPS > C); expression of DNA repair genes OGG1 and XRCC (WPS < CS < C); expression of carcinogen activation/metabolism gene CYP1A1 (WPS > CS > C); expression of detoxifying enzyme genes (NQO1 - CS < WPS < C; GSTA1 - WPS < CS, C).	Self-reported exposure only; convenience sample; no control for confounding.
Alsatari et al.^96^ 2012	Cross-sectional	N=68 (50 WPS, 18, CS, 18 C); 26.5±4.2 years for WPS, 25.2±5.4 for CS, 26.3±7.6 for C; 100% male; Irbid City, Jordan	WP smoking; WP frequency (high: ≥1 session/day; medium: 4–5 per week; low: <3 per week)	Systemic genotoxicity	Chromosomal aberrations (CA) chromatid and chromosome gaps and breaks and exchange – WPS (3.7x) > CS (2.7x) > C; CA – WP high > WP medium > WP low	Self-reported exposure; participant selection unspecified; participants matched on age, exclusion of persons with alcohol and/or drug use.
Eker et al.^22^ 2016	Cross-sectional	N=60 (30 WPS and 30 C); 18–25 years; sex distribution of both groups were similar; Turkey	WP smoking	Systemic genotoxicity	Chronic: no statistically significant difference between the WPS and C groups in terms of chromatid and chromosome breakages; WPS > C for fragments and gaps.	Self-reported exposure; participant selection approach not stated; no stated control of confounding.
Hawari et al.^42^ 2019	Cross-sectional	N=738 (135 WPS, 303 CS, 300 C); 18–27 years (mean: 21.5±1.8 for WPS, 21.7±1.9 for CS, 21.6±1.9 for C); 100% male; college campuses in Egypt, Jordan, Morocco, Oman	WP smoking, WP duration, WP frequency	Quality of life (evaluated using Short Form 12)	WPS or CS > C for general health score; WPS > C for emotional limitation domain score.	Self-reported exposure; self-reported outcome; convenience sample; controlled for environmental exposures, BMI, physical activities, excluded persons with chronic diseases.
Khabour et al.^97^ 2011	Cross-sectional	N=86 (50 WPS (18 heavy, 16 medium, 16 light), 18 CS and 18 C); 28.3±2.1 years for WPS heavy, 26.1±1.7 years for WPS medium, 24.9±1.8 years for WPS light, 25.2±1.4 years for CS, amd 26.2±1.8 years for C; 100% males; Irbid city/Jordan	Frequency of WPS per week	Systemic genotoxicity (in lymphocytes)	Frequency of sister chromatid exchange – WPS heavy > CS > C; WPS heavy > WPS medium > WPS Light > C. Mitotic index: WPS + C > C but not statistically different.	Self-reported exposure; convenience sample; no control for confounding.
Khalil et al.^98^ 2019	Cross-sectional	N=50 (25 WPS group and 25 C); Age range between 18–25 years; gender distribution not provided but reported to be similar for both groups; Philadelphia, Jordan	WP smoking (no cigarette but smoke WP for more than 3 times per week for more than 2 years)	Systemic genotoxicity (in blood cells)	WPS > C for chromosome breakage, fragments, and gaps.	Self-reported exposure; convenience sample; stated that gender distribution and sample size were similar among both groups but no control of potential confounding factors otherwise.
Muddathir al.^94^ 2018	Cross-sectional	N=120 (40 WPS, 40 CS, 40 C); WPS 18–48 years (mean: 27.8±3.9); CS 18–47 years (mean: 30.1±5.2); C 19–51 years (mean: 29.6±4.5); Khartoum, Sudan	WP smoking, WP frequency and duration	Systemic – coagulation factors fibrinogen, factor VII and factors VIII	WPS > CS > C for fibrogen, factor VII and factors VIII; fibrinogen and factor VIII greater in WPS for WP use >3 years vs ≤3 years	Self-reported exposure; convenience sample; excluded individuals with history of platelet abnormalities, bleeding or vascular disorders, liver or renal disease, medication affecting platelet function.
Rajab et al.^99^ 2019	Cross-sectional	N=207 (88 WPS, 119 C) 18–25 years. 100% female; Damascus, Syria	WP smoking	Systemic – folate levels Systemic inflammation (hs-CRP levels)	Folate median WPS < overall group hs-CRP levels not different between smokers and non-smokers.	Self-reported exposure to WP; convenience sample used; no control for confounding.
Strulovici-Barel et al.^33^ 2016	Cross-sectional	N=40 participants (19 non-smoker C and 21 WPS); all participants ≥18 years (mean: 33±9 years in C group and 25±4 years in WPS group); 90% male in C, 65% male in WPS group; 6/5/8 black/white/other in C group and 8/2/11 black/white/other in WPS group; New York City, USA	WPS smoking	Systemic – plasma endothelial microparticles (EMPs)	WPS > C for total EMP	Self-reported exposure verified with cotinine and nicotine measurements; convenience sample used; no confounder control but study participants in C.
Yalcin et al.^36^ 2017	Cross-sectional, pre-post	N=100 (50 WPS/WPS + CS and 50 C) 18–38 years (26.72±5.2 years for WPS, 27.46±5.3 years for C); 66% male for WPS, 64% male for C; Ankara, Turkey	WP smoking; WP duration; exhaled CO	Systemic oxidative stress	After smoking, total oxidant and total antioxidant statuses and oxidative stress index higher, and salt-stimulated paraoxonase activity lower in WPS.	Controlled WP smoking session; objective exhaled CO used to quantify exposure but association with outcome not analyzed; convenience sample used; no control for confounding.

*WPS: waterpipe smokers. CS: cigarette smokers. C: control.

#### Psychological effects

No association was observed for depressive symptoms, anxiety, or stress, in three studies of college students^78,81,82^. In contrast, the odds of past 30-day WP smoking increased by 30–140% with self-reported mental health diagnosis (including depression, anxiety, sleeping disorder, attentiondeficit disorder, and addictive disorder) in a national survey of college students^80^. The odds of current use of WP was also associated with a psychological stress scale (on Cohen’s Perceived Stress Scale) and a depression scale (the Center for Epidemiologic Studies Depression Iowa Short Form) but not with self-reported mental health diagnosis in another study of college students^83^. Marsden et al.^79^ observed that the score on the Center for Epidemiologic Studies Depression 10 Scale increased by 3% with every 5-day increase in usage in the past 30 days in their study. Also, the score increased by 4% and 9% for at least 5 days and 15 days of usage in the past 30 days, respectively, among college students^79^. Unlike the other studies, the results from the Marsden et al.^84-86^ study were based on longitudinally collected and repeated measures data with some information about directionality. They were also able to adjust for the use of other tobacco products in their analyses.

#### Neurocognitive effects

A few studies have examined the association between WP smoking and cognitive function in young adults. There was no difference in the assessed cognitive function between WP smoking and non-smoking male young adults before and immediately after supramaximal exercise in one of the studies^84^. The complexity of the cognitive test (number-recall) that was administered in the study may have been insufficient to detect a difference, and the sample size (10 per group) was small. However, the two-hands coordination, attention and concentration, reactive stress tolerance and reaction speed parameters on the Vienna Test System’s traffic test battery improved after WP smoking among male college student participants in a pre-post study^85^. This effect was theorized as being due to the acute enhancement of fine motor performance, alerting attention accuracy, and response time, by nicotine. The potential chronic effect of WP smoking on these driving test parameters was not tested since the study did not include non-smoking controls. On the other hand, Meo et al.^86^ reported significant decline in attention switching, complex reaction time, and pattern recognition memory variables on the Cambridge Neuropsychological Automated Battery (CANTAB) test among WP smoking young adults compared to non-smoking controls.

Notwithstanding the inconsistencies in the results of the aforementioned studies, decreased circulating brain-derived neurotrophic factor (BDNF) among middle school adolescent smokers (vs non-smokers) indicate the potential for adverse effect of WP smoking on mental health at a young age^87^. BDNF is a neurotrophin that is important for neural development and synaptogenesis, and plays a key role in learning and memory^87,88^. Consequently, a decline in its circulating concentration, which is correlated with BDNF levels in the brain, might be expected to result in cognitive and behavioral deficits^87^.

#### Comments about studies of neurological and psychological effects

It is unique that all the studies of the association between WP smoking and mental disorders were conducted in the US. All the studies of mental health were large population-based studies that relied on self-reported smoking status information. The four studies on cognitive and potential neurological effects were conducted in the Middle East, and three of these included only male participants. However, none but one evaluated the effect of gender or race on the associations between WP smoking effect and the outcomes. Primack et al.^7^ reported higher odds of WP smoking in college women versus men with addictive disorders.

### General health and systemic effects

WP smoke contains considerably larger amounts of CO than cigarette smoke^9^. As would be expected, multiple cases of CO poisoning due to WP smoking among adolescents and young adults have been reported in the literature (Table 5)^89-92^. Also, WP smoking induces systemic oxidative stress and inflammatory responses (Table 5)^36,56,93-95^. In one study, total oxidative status and antioxidant status, and their ratio (oxidative stress index), were increased by 205%, 15%, and 180%, respectively, in young adults following WP smoking compared to levels in non-smokers, whereas salt-stimulated activity of antioxidant enzyme paraoxonase was correspondingly reduced^36^. However, the baseline pre-smoking levels of these biomarkers were not measured in the WP smokers. The expression of the xenobiotic-detoxifying enzymes NAD(P)H:quinone oxidoreductase 1 and glutathione S-transferase A1 in peripheral blood were also reduced in WP smokers compared to non-smoking controls in another small study (15 per group) comprised mostly of young adults^93^.

Increased oxidative DNA damage in peripheral blood and lymphocytes has been reported in WP smoking young adults^96-98^. While no difference was observed in one study^21^, increased chromosomal aberrations including chromosome and chromatid gaps and breaks, sister chromatid exchange and/ or chromosome fragments in blood cells were reported among young adult WP smokers in all other studies^96-98^. Chromosomal aberrations were about 2x and about 4x more likely among those that used WP at least once a day compared to those who smoked 4–5 and <3 times per week, respectively^96^. Additionally, increased biomarker of DNA damage, 8-hydroxy-2’-deoxyguanosine (8-OHdG), but reduced expression of DNA repair genes including oxoguanine glycosylase 1 and X-ray repair cross complementing 1 protein, were observed in the blood of WP smokers compared to non-smoking controls^93^. The chromosomal aberrations were even more increased in WP smokers compared to cigarette smokers, which aligns with the higher yields of mutagenic and carcinogenic compounds in WP smoke^96^. In contrast, the blood concentration of 8-OHdG was higher in cigarette smokers, but this may be due to the reduced expression of DNA repair genes among WP smokers compared to cigarette smokers^93^. Furthermore, a dose-response relationship was observed between sister chromatid exchange and the frequency of WP smoking^97^.

Based on its oxidative effects, it is expected that WP smoking would induce systemic inflammation in adolescents and young adults. Nonetheless, no cross-shift change was observed in circulating proinflammatory cytokines interleukin-1β (IL-1β), IL-6 and IL-8 among WP bar workers^56^, and the blood concentration of acute phase C-reactive protein was similar in female WP smoking and non-smoking university students^99^. However, interferon-γ and tumor necrosis factor-α increased across the work shift among the bar workers^56^. Furthermore, there is an apparent effect of WP smoking on biological processes that are regulated by proinflammatory cytokines, including coagulation and angiogenesis^100,101^. Coagulation factors, fibrinogen, and factors VII and VIII, were increased in WP smokers compared to cigarette smokers and non-smokers with the levels of fibrinogen and factor VIII being associated with the duration of WP usage^94^. In contrast, vascular endothelial growth factor (VEGF), an angiogenic factor, was reduced in adolescents who smoked both cigarettes and WP, and those who smoked WP exclusively, compared to non-smoking controls^95^.

The effect that was associated with exclusive WP smoking was due to differences in boys and not girls. Although, the relationship between tobacco smoking and VEGF is inconsistent in the literature, Alomari et al.^95^ hypothesized that the increased amounts of CO and polycyclic aromatic hydrocarbons in WP smoke may inhibit the production and/or enhance the depletion of VEGF mRNA and protein in blood vessels. Finally, folate was reduced among female WP smoking university students compared to nonsmoking controls^99^, while WP smoking male young adults scored lower on the general health and emotional limitation domains of the 12-item short form quality of life survey^42^.

#### Comments about studies of general health and systemic effects

All but three of the studies reporting on the general health and systemic effects of WP smoking were conducted in the Middle East^56,90,92^. All were either case studies or cross-sectional in design. While some included both male and female participants^21,56,89,92,95^, only one reported results about differences in the effect of WP smoking by gender^95^. Dose-response information was reported for only two of the studies^94,97^.

## CONCLUSION

As expected in studies of adolescents and young adults, subclinical effects were the most investigated outcomes. Notwithstanding the weaknesses in the studies, results were consistent for the genotoxic effects of WP smoking which were observed to be more potent than cigarette smoking in a few studies. Similarly, results were consistent for effects on lung function and in the oral cavity. As reported in case studies, WP smoking can also result in acute clinical cases of lung injury and infection, and systemic CO poisoning. The plausibility of these effects, which are also reported for cigarette smoking, is obvious as WP smoke contains many of the same toxic components of cigarette smoke, and many of these in much larger amounts (e.g. particulate matter, CO, polycyclic aromatic hydrocarbons, and semi-volatile furans). Furthermore, these effects are involved in the pathogenesis of local and systemic chronic diseases (Table 6). Alteration of saliva biochemistry including its pH, buffering capacity, and the oral microbiome, increases the risk of tooth decay and oral infection^18,19^, while decline in lung function (spirometry measures) above age-related decrease and adverse changes in cellular composition in the small airways of the lungs, which are indicative of impairment in clearance mechanisms, contribute to the development of chronic lung diseases^33,39^. Also, chronic hematological changes, elevated BP, impaired vascular function, and altered cardiac autonomic function can be induced by continuous exposure to exogenous insults and are risk factors for cardiovascular disease^59,63,76,77,84^. Finally, chronic induction of genotoxicity, including damage to the DNA and chromosomes and deleterious epigenetic modification, is integral to the development of cancer^21,25,32,33^. Although asthma and type 2 diabetes are associated with cigarette smoking in young adults^102-105^, studies of the relationships between WP smoking and both diseases are lacking. Nonetheless, findings from two cross-sectional surveys of high school students in Florida suggest that there might be an association with asthma^50,51^.

**Table 6:** Implications of related subclinical physiological changes that are observed in relation to WP smoking in adolescents and young adults for chronic disease pathology

*Outcome measures*	*Implication/meaning*
Alteration of saliva biochemistry	Changes in the acidity (pH) and ability to buffer against it in the saliva can cause loss of enamel, increase potential for infection and disease (e.g. oral thrush). Alteration of background microorganism composition (microbiome) in the oral cavity as observed for WP may increase the risk of infection^18,19^.
Spirometry or lung function	Decline in lung function was often associated with WP smoking in the studies. Rapid (non-age related) decline in lung function (volume capacity and air flow from the lungs) is involved in the pathogenesis of respiratory diseases including asthma, chronic obstructive pulmonary disease (COPD), and lung cancer^39^.
Cellular composition in small airway epithelium	The decrease in ciliated cells and increase in mucous secreting cells in small airway epithelium degrades the lung clearance mechanism. Such alterations can be caused by exogenous insults, were observed in relation to WP smoking, and do occur during the development of chronic respiratory diseases^33^.
Hematological parameters	WP increased hematological parameters including hematocrit (proportion of red blood cells in the blood) and white blood cell counts. Chronic changes in these parameters alter cardiovascular function and may contribute to or indicate disease^84^.
Hemodynamic measurements	Measures of blood flow dynamics including BP are altered by WP smoking. An overly elevated BP (hypertension) or reduced BP (hypotension) may precipitate or indicate chronic diseases including in the cardiovascular system^63^.
Vascular function measures	Measures of vascular function including FMD, arterial stiffness, and pulse wave velocity indicate the stiffness and reactivity of blood vessels. Changes in vascular function is an early indicator of atherosclerotic cardiovascular disease and is induced by WP smoking^59,76^.
Cardiac autonomic control	Change in the autonomic control of cardiovascular function is a predictor of coronary heart disease and mortality and is induced by WP smoking^60,77^.
Brain-derived neurotrophic factor ( BDNF )	BDNF is involved in brain function and homeostasis. Reduced circulating BDNF concentration in the blood was observed in association with WP smoking and may result in cognitive and behavioral deficit in the long-term^87^.
Genotoxicity	This includes damage to the structure of the DNA and chromosomes. Genotoxicity initiates the cancer mechanism and its continuous induction by exogenous insults, similar to the observations for WP, can overwhelm cellular defence mechanisms and increase cancer risk^21,25^.
Epigenetic modification	These are modifications on the DNA or histone that do not change the DNA sequence. They may affect gene transcription and associated expression (production) of protein. Deleterious epigenetic changes, such as was observed in relation to WP smoking, adversely alter cellular metabolism and are often involved in the pathogenesis of chronic diseases including cancer and COPD^32,33^.
Oxidative stress and inflammatory measures	Oxidative stress (imbalance between production of oxidant species and antioxidant defences in the body) and inflammation (triggering of the immune response) are involved in the pathogenesis of chronic inflammatory diseases (e.g. cardiovascular disease, COPD, and cancer) and are both induced by WP smoking.

While 65 studies met the criteria for selection in this narrative review, the literature on this topic is still quite nascent. Most of the studies are case studies, cross-sectional, or pre-post in design; one is a crossover study while only two are longitudinal in design. Most relied on convenience samples, included only male or only female participants, and/or relied solely on selfreported smoking as the exposure metric. Therefore, information about exposure–response relationships and differences by demographic factors are mostly lacking. Also, there is a dearth of information about the effect of WP smoking among young adults in Western countries such as the US, the demographic group experiencing the greatest growth in prevalence of WP use. However, confounding of the associations of outcomes with WP usage, due to the smoking of alternative tobacco products, was mitigated in most of the studies. Twenty-two of the selected studies were case or pre-post studies that investigated outcomes following WP use, while smoking more than one tobacco product was an exclusion criterion, or controlled for, in 31 of the remaining 43 studies.

To comprehensively understand the effect of WP smoking, the generalizability of findings must be improved by conducting more studies outside the Middle East, in countries where there has been a recent rapid uptake of WP smoking among adolescents and young adults. In addition, future research should include sufficient sample sizes of participants distributed equally among the sexes so that gender differences can be explored, should be longitudinal in design, and should sufficiently control for confounders including the objective measurement of tobacco smoking, such as urinary or salivary cotinine. The results from such studies will underpin the development of effective regulations and effective educational campaigns designed to curb WP smoking among young adults.

## Data Availability

Data sharing is not applicable to this article as no new data were created.
